# Chatbots for future docs: exploring medical students’ attitudes and knowledge towards artificial intelligence and medical chatbots

**DOI:** 10.1080/10872981.2023.2182659

**Published:** 2023-02-28

**Authors:** Julia-Astrid Moldt, Teresa Festl-Wietek, Amir Madany Mamlouk, Kay Nieselt, Wolfgang Fuhl, Anne Herrmann-Werner

**Affiliations:** a University of Tuebingen, Tuebingen, Germany; b Institute for Neuro- and Bioinformatics, University of Luebeck, Luebeck, Germany; c Institute for Bioinformatics and Medical Informatics, University of Tuebingen, Germany; d Department of Internal Medicine VI/Psychosomatic Medicine and Psychotherapy, University Hospital Tuebingen, Tuebingen, Germany

**Keywords:** Medical students, artificial intelligence, applications in education;, human-computer interface, teaching/learning strategies, chatbot

## Abstract

Artificial intelligence (AI) in medicine and digital assistance systems such as chatbots will play an increasingly important role in future doctor – patient communication. To benefit from the potential of this technical innovation and ensure optimal patient care, future physicians should be equipped with the appropriate skills. Accordingly, a suitable place for the management and adaptation of digital assistance systems must be found in the medical education curriculum. To determine the existing levels of knowledge of medical students about AI chatbots in particular in the healthcare setting, this study surveyed medical students of the University of Luebeck and the University Hospital of Tuebingen. Using standardized quantitative questionnaires and qualitative analysis of group discussions, the attitudes of medical students toward AI and chatbots in medicine were investigated. From this, relevant requirements for the future integration of AI into the medical curriculum could be identified. The aim was to establish a basic understanding of the opportunities, limitations, and risks, as well as potential areas of application of the technology. The participants (*N* = 12) were able to develop an understanding of how AI and chatbots will affect their future daily work. Although basic attitudes toward the use of AI were positive, the students also expressed concerns. There were high levels of agreement regarding the use of AI in administrative settings (83.3%) and research with health-related data (91.7%). However, participants expressed concerns that data protection may be insufficiently guaranteed (33.3%) and that they might be increasingly monitored at work in the future (58.3%). The evaluations indicated that future physicians want to engage more intensively with AI in medicine. In view of future developments, AI and data competencies should be taught in a structured way during the medical curriculum and integrated into curricular teaching.

## Introduction

The healthcare system is undergoing a digital transformation, and artificial intelligence (AI) will play a significant role in defining everyday medical practice [1]. The location- and time-independence of digital applications have created new opportunities for medicine and health communication that are also changing the doctor – patient relationship [2]. The growing importance of e-health applications, wearables and AI applications such as chatbots can empower patients to collect their own health data [3,4].

Furthermore, the digital networking of patients, hospitals, physicians and other healthcare services is enabling a shift from a physician-centric approach to more patient-centered treatment [5]. To exploit the potential of this technical innovation and ensure optimized care for patients, future doctors must be equipped with the appropriate skills [6]. Future physicians will not only need to be flexible in responding to different healthcare contexts but will also require the competence to adequately deal with procedures and applications involving AI and the accompanying big data [7]. The growing complexity of medicine and increasing specialization of knowledge require the integration of AI as well as the interaction with digital assistance systems already in the curriculum of medical studies [8–10]. According to current literature, although AI competencies are essential for medical practice, they are not comprehensively taught in medical education [7,11,12].

### Medical curriculum in Germany

A look at the national competence-based learning objectives catalog for medicine (NKLM) [13] shows that the teaching of competencies in the area of medical apps and artificial intelligence is still underrepresented. The national competence-based learning objectives catalog for medicine is currently being further developed on the basis of the ‘Master Plan for Medical Studies 2020’ [14]. It is to be made compulsory at all medical faculties when the new Medical Licensing Regulations come into force. The focus is on the question of which competencies junior doctors should acquire as part of the core curriculum of their medical studies, including medical communication skills, interprofessional teamwork, scientific work and digital competencies [15]. The importance of integrating AI into medical studies is already being explored extensively in research and literature, but curricular implementation [16,17] is in its early stages. The emerge of medical chatbots could have the potential to improve the efficiency, quality and accessibility of healthcare services by providing quick information and connecting patients to healthcare providers and will become increasingly important for both doctors and patients during the treatment process. However, there are also raising concerns about chatbots potentially eroding diagnostic practice, being driven by business logic, and having limitations and incompleteness that may harm patients in emergency situations [18,19]. Understanding how these technologies work and how they can be used can help medical students better understand the broader landscape of healthcare and be better prepared for the future of medicine [20]. As far as the authors know, the experience and attitude towards the use of chatbots in medicine among medical students has not yet been recorded in Germany.

Therefore, the question arose for us, how to successfully prepare medical professionals for this new scenario during their medical training. This study is intended as basic research relating to AI in the medical curriculum. We aimed to provide an overview of the current knowledge and attitudes of medical students regarding AI technologies to determine what is necessary for future implementation in the curriculum. Therefore, the following questions arose regarding the implementation of AI in medical education:

- What attitudes do medical students have about artificial intelligence and the use of chatbots in medicine?
- Does their view of chatbots change after learning more about medical chatbots in the course?

## Material and methods

### Study design and participants

This study followed the Standards for Reporting Qualitative Research [21] and presents a mixed-method study as part of a project supported by the Federal Ministry of Education and Research, Germany (BMBF). It was carried out at the Medical Faculty of Tuebingen with the support of the Institute for Neuro- and Bioinformatics, University of Luebeck. A hybrid course named ‘Chatbots for Future Docs’ was developed for medical students of all semesters and was offered as an elective course between January and March 2022. *N* = 12 medical students learned about conditions of doctor – patient communication in general, possible uses of chatbots in healthcare, the ethical framework of AI, how chatbots work in general and what it takes to create a ready-to-go bot. The course combined classical teaching of theoretical knowledge (represented by asynchronous digital short inputs) and practical competence acquisition in the form of exercises and group work during a synchronous online format via Zoom.

The attitudes of participants regarding the topic of AI in medicine were quantitatively assessed by means of a standardized questionnaire before and after the course via the learning management software ILIAS. Qualitative methods such as group discussions supplemented the detailed, subjective and individual attitudes of the medical students to the topic.

#### Ethics

The study received ethical approval from the Ethics Committee of Tuebingen Medical Faculty (no. 824/2021BO2). Participation was voluntary, and students provided their informed consent and received no compensation. All responses and data were kept anonymous.

### Measurement and process of study

We combined quantitative and qualitative research methods in this study to achieve an in-depth analysis of the collected data [22].

To conduct the quantitative survey, the medical students were asked to complete a self-developed standardized questionnaire at the beginning of the course to assess subjective attitudes toward AI in medicine. The questionnaire was divided into three subsections, with items including five-point Likert scales and multiple-choice questions. The first subsection aimed to gather demographic data including age, gender and general attitudes toward technology and AI. Attitudes toward technology use were assessed using a shortened version of the validated short scale for assessing individual differences in the willingness of technology, as per Neyer et al. [23] (technology commitment). The second subsection examined the openness of medical students regarding the future use of AI in healthcare in particular, which in the future may be able to answer health-related questions, perform certain tests or examinations, diagnose health conditions and suggest and apply treatments. The third subsection addressed the attitudes of the medical students toward chatbots in medicine. Students were asked to revisit two questions from the questionnaire on chatbots in medicine after the course, as this was the main interest of the course.

The material for qualitative content analysis was based on group work conducted by the students, which involved discussing questions about the use of mental health chatbots in medicine (see A1, appendix).

### Data analysis

#### Quantitation

Statistical analysis of the questionnaire was performed using IBM SPSS version 27. Mean values (M), standard deviations (SD), sum values, frequencies and percentages of the relevant items were obtained. The Pearson correlation coefficient was calculated to capture linear relationships between the relevant variables. We used the Wilcoxon test to analyze the statistical significance of the differences between the two repeated items after the course; the level of significance was *p* < .05.As one student did not complete the course, we used the mean imputation procedure for the missing values after testing for normal distribution using the Kolmogorov-Smirnov test procedure [24,25].

#### Qualitative analysis

The results of the students’ group work were recorded, transcribed and coded by three different authors (JAM, TFW, AM). Following discussions in regular meetings, findings were summarized and a category system consisting of main and subcategories (according to Mayring’s qualitative content analysis) was agreed upon [26]. Selected text passages were used as quotations to illustrate each category [26]. Inductive category formation was performed to reduce the content of the material to its essentials (bottom-up process). In the first step, the goal of the analysis and the theoretical background were determined. From this, questions about chatbots in medicine were developed and presented to the students for discussion in a group exercise. Two main topics were identified, namely positive and negative attitudes toward chatbots in medicine. In the second step, we worked through the individual statements of the students systematically and derived various categories: user group, technical implementation, acceptance, and use in medicine.

## Results

The course was attended by 12 medical students from the clinical and preclinical study sections, who were able (at least partly) to give a broad explanation of AI. For further information, please see Table 1.

**Table 1.** Students’ previous knowledge about AI and demographic data.

Yes, I could explain the main features and applications of AI	6/12 (50.0%)		
I could give a general explanation, but I don’t know anything more specific than that	6/12 (50.0%)		
Age	Mean 24.8	Standard deviation 2.9	Min/Max 21/29
Gender	Male 5 (41.7%)	Female 6 (50.0%)	Other 1 (8.3%)

### Main results questionnaire

#### Part 1: general attitudes toward technology, and socio demography

The participating students stated that they could explain the main features and applications of AI (50.0%) and that they could at least give a broad definition of AI (50.0%). Evaluation of the technology commitment according to Neyer et al. 2016 showed that the study participants displayed a moderate willingness to use technology (*M* = 21; SD = 3.1, min. 18, max. 31).

#### Part 2: attitudes toward AI in medicine

**Figure 1.**
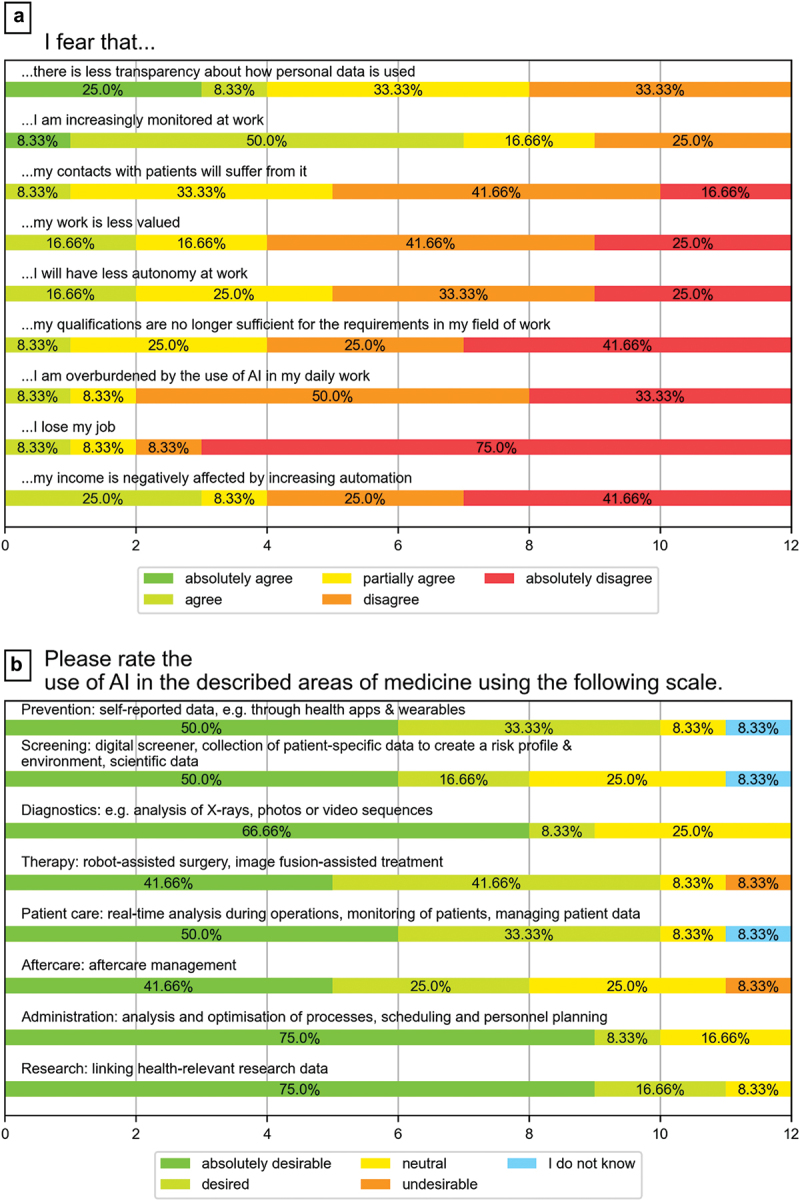
Attitudes of medical students toward AI in medicine (fears about AI in various areas of medicine).

Many students were not afraid of the future changes that AI could bring. The majority (83.3%) were not afraid of losing their jobs because of AI or of being overwhelmed by using AI (83.3%). However, some students agreed that they were afraid of increasing surveillance at their future workplaces (58.3% agreement vs. 25.0% disagreement) and decreasing transparency regarding the use of personal data (33.3% agreement vs. 33.3% disagreement) (Figure 1 Plot A).

As shown in Figure 1 Plot B, the majority of students supported the use of AI in various areas of medicine. Nevertheless, there was less support for its use in human-centered areas such as therapy or follow-up treatment, while the use of AI in more technical areas such as diagnostics or administrative tasks was seen as mostly beneficial.

**Figure 2.**
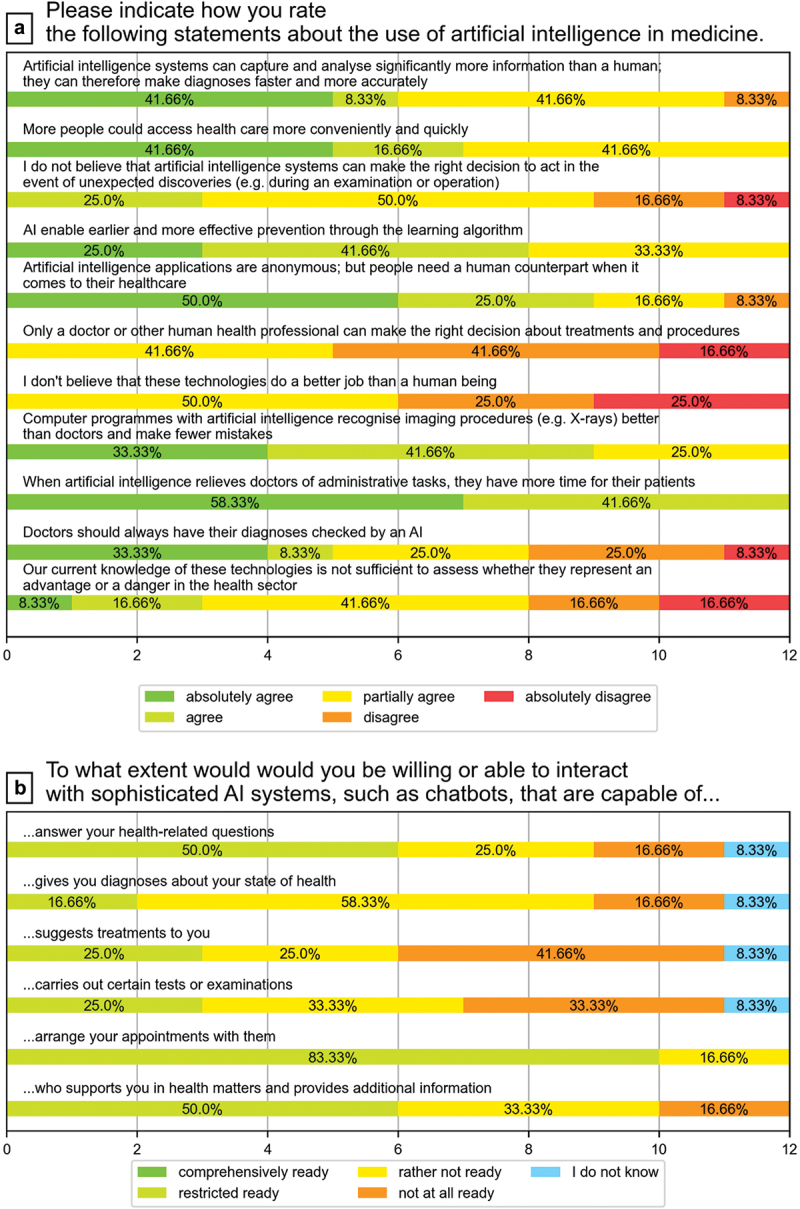
Attitudes of medical students toward AI in medicine (statements about the use of AI and chatbots).

Students saw the use of AI in medicine primarily as an opportunity to reduce the administrative burden on physicians. At the same time, however, they also believed that the new technology would be able to make diagnoses faster and more accurate in the future and ultimately also make access to medical advice more sustainable (Figure 2 Plot A).

#### Part 3: attitudes toward chatbots in medicine

At the beginning of the course 692% of the students never have used a medical chatbot before. However, the students had great confidence in chatbots, especially for organizational tasks. In task areas with high levels of responsibility, such as diagnosis or treatment suggestions, opinions tended to diverge and were more critical, but the students were not completely opposed to the use of AI in general (Figure 2 Plot B).

As shown in Tables A2 and A3 in the appendix, no major differences in attitudes held before and after the course were detected. However, students were significantly (*p* = 0.02) less critical about privacy and the use of medical chatbots after the course. Prior to the course, 500% of students believed that data privacy and security could not be fully guaranteed, 41.7% were undecided, and only 8.3% did not believe this. After the course, only 36.4% were still critical of data protection, 18.2% were undecided and 45.5% did not believe that data protection cannot be guaranteed with med. Chatbots (see Table A3). Only a few statements showed a clear positive or negative tendency. For example, the majority agreed that using chatbots saved time (83.3% agreement pre; 72.7% agreement post) and money (75.0% agreement pre; 54.5% post). However, medical students felt that chatbots were not yet sufficiently established and that long-term success had yet to materialize (81.8%). There was also a fear of communication problems (81.8%) and loss of personal contact with patients (63.6%) because of the lack of maturity of the technology.

### Findings of the qualitative content analysis

The main findings of the present study concern the students’ views on chatbots in medicine. Four main themes/categories were identified: user group, technical implementation, acceptance, and use in medicine (Table 2).

**Table 2.** Overview of categories, subcategories and corresponding examples based on qualitative analysis.

category	Subcategory	Example
User group	Positive Attitudes	Enabling of individual chat selection (in case someone needs small talk or similar) Possible individual language selection Target group-oriented language possible
	Negative Attitudes	Specific user groups are not confident with modern techniques and chatbots
Technical implementation	Positive Attitudes	Communication tools, e.g., emojis and artificial delays could be helpful to simulate human-like conversation
	Negative Attitudes	By now, algorithms are too strict to enable flexible and individualized answer design Smalltalk is not expedient
Acceptance and trust	Positive Attitudes	Anonymity reduces reluctance and shame when disclosing personal and hurtful information
	Negative Attitudes	Skepticism regarding the ability of the intermediary instance chatbot to support physicians as this could limit the direct doctor–patient relationship Informational gain is questionable due to lost information from nonverbal communication Initial personal talk remains indispensable Data security
Use in medicine	Positive Attitudes	Reflecting through chatting as a therapeutic tool but not as a substitute for therapy Interactive diary
	Negative Attitudes	Critical toward chatbots as a therapy tool Human contact and empathy are crucial for therapeutic success

#### Theme 1: user group

The medical students were positive that chatbots are accessible to a broad user group as a result of their time- and location-independent availability. They also believed that possible language barriers or other hurdles could also be overcome. Nevertheless, they were concerned that certain groups of people (elderly people, visually impaired people) may not be familiar with modern technology or chatbots. The medical students also proposed a minimum age of 18 years and an alternative language mode specifically aimed at children.

#### Theme 2: technical implementation

The students felt that with the use of emojis, artificial delays, small talk and customization, there was an opportunity to provide the most human-like and realistic conversation possible with a chatbot. Furthermore, the development of the chatbot should take into account the context in which it would be used (administrative or personal assistant). However, according to the students’ evaluations, technical implementation was not sufficiently mature or flexible because of the limited and rigid algorithms used.

#### Theme 3: acceptance and trust

The use of chatbots as therapy tools or interactive diaries for patients was considered by the students to be a good opportunity to reduce anonymity, shyness and shame about disclosing personal and painful information. However, it was thought that doctor – patient communication could deteriorate as a result of non-verbal communication with a chatbot and interpersonal information may get lost. Thus, the first personal conversation between doctor and patient was considered indispensable.

#### Use in medicine

The students perceived the use of therapy chatbots in medicine as potentially supportive and complementary diagnostic tools that could be used independently by patients but did not understand them as a substitute for therapy. As an example, the students mentioned the possibility of technical errors or the risk of increasing social withdrawal in depressed persons due to the lack of personal contact. Therefore, human contact and empathy were considered essential for the success of therapy, which could be reduced by the use of chatbots.

### Teaching evaluation

The participants enjoyed gaining a fundamental insight into the topic of chatbots and AI, which was completely new for some of them. In addition, the interactive design and hybrid format contributed to the positive evaluation of the course. In general, the students gained a new perspective on chatbots and their associated problems, as well as a basic understanding of how chatbots function, where they can be used and the effort involved in implementing them.

## Discussion

In the context of digitalization in healthcare, applications that use AI are becoming increasingly important [12]. This study contributed to the understanding of the current level of knowledge and attitudes regarding AI, particularly chatbots, in medicine.

Medical chatbots are an example of how artificial intelligence and technology are being integrated into healthcare. As such, it would be beneficial for medical students in Germany to learn about this technology as part of their medical curriculum. This will help ensure that they are well-equipped to work with and utilize medical chatbots in their future practice, in order to provide high-quality and efficient care to patients. Additionally, learning about medical chatbots would provide medical students with a better understanding of the role that technology plays in healthcare and how it is likely to continue to shape the medical profession in the future.

This study revealed that the attitudes and expectations of medical students were generally optimistic about the use of AI and chatbots in relation to a variety of purposes in medicine, including in areas such as administration, research and diagnostic imaging techniques (Figure 1, Plot B). In particular, the students would trust the chatbots to perform recurring and supportive activities, such as answering simple questions, arranging appointments, and providing basic information. The majority were certain that they would not be replaced by AI in their jobs or that they would be less valued in their roles as future doctors, as other authors have stated in similar studies [27]. However, they were more critical of the use of these new technologies in core tasks, such as carrying out personal counseling and specific medical examinations. The participants also expressed concerns that data protection and privacy may no longer be adequately guaranteed. Finally, there was a fear that personal contact with patients could be lost if the patients were increasingly engaging with technical systems rather than human personnel.

As other studies have demonstrated, it cannot be assumed that the generation of people who have grown up with digital technologies and are proficient in their use (similar to our cohort) are also aware of all the options and ethical consequences of the use of new technologies in their professional field. However, this is not synonymous with the simultaneous development of digital competencies in the professional field [28,29]. The areas in which AI can be applied in medicine are diverse and, with the development of smartphone apps, have reached not only the healthcare system but also the private sphere – and there will likely be more in the future [30]. Accordingly, to remain empowered, future physicians must be able to understand how AI works, as well as how to interpret results, in order to meaningfully support patients with digital tools at the same time as critically monitoring AI [31]. Digital learning opportunities and the development of AI skills are essential in medical education and may help to meet the vast need for qualifications [32]. We found a great deal of uncertainty and skepticism regarding chatbots due to the lack of integration of AI topics into the medical curriculum (as yet), as well as a lack of knowledge about the basic conditions and legal and ethical requirements of AI use [33], reflecting findings from other studies [34–36].

While students saw a great deal of potential for the use of chatbots in medicine, they had many concerns about using them in areas that went beyond organizational activities such as making patient appointments. Above all, they believed that the technology was not yet sufficiently developed and that in the context of healthcare, patients needed a human counterpart. Neutral attitudes to chatbots were also evident from many statements in the questionnaire, which confirmed the thesis that there is not yet enough knowledge about the topic of AI in medicine for the study participants to have developed distinct opinions. In the questions about chatbots, in particular, which were repeated after the course, we could not identify any significant changes in attitudes. However, our study was able to give medical students, as non-computer scientists, a good initial overview of how a chatbot works, a basic understanding of how much data needs to be provided for an AI, and also potential future uses of AI in medicine and medical chatbots.

The perceived challenges and concerns of students relating to the use of AI and chatbots in healthcare must be addressed and taken seriously before future physicians are exposed to such tools [37]. AI is still underrepresented in the medical curriculum, and students lack the opportunity to engage more intensively with the topic of AI and develop the required expertise [11,38,39]. Therefore, for us, it was important not only to teach digital competencies and knowledge about AI theoretically but also to incorporate them practically into the teaching unit. Thus, digitization was both included in the teaching and incorporated as a learning objective.

### Limitations

Although the students were very interested in the topic of chatbots in medicine, and the topic of AI is also gaining increasing importance in medicine in general, the results of our study are limited in terms of representing the perspectives of the student population due to the small number of participants. Also, the course duration of three months was too short for sustainable changes in viewpoints or information gain to occur. However, the goal of this work was not to draw representative conclusions for all German medical students, but rather to understand the state of knowledge and perceptions of medical students regarding AI and chatbots in medicine, which we believe was achieved with our sample. The next step will be to investigate which AI competencies should be included in the curriculum.

## Conclusion

This study indicated that future physicians in Germany are willing to engage more intensively with AI in medicine. In our study, they were able to develop a basic understanding of how AI and chatbots will affect their future daily work. Although their basic attitude toward the use of clinical AI was positive, medical students also had concerns, especially with regard to the lack of data protection and declining personal contact with patients. With a view to future developments in the workplace, we can only emphasize once again how urgently medical curricula need to be supplemented with these new core competencies so that doctors can help to shape the technological course of patient treatment in an informed and self-confident manner.

## Appendices

### A1. Questions from the group work about mental health chatbots in medicine

- How would you rate chatbots as therapy tools, for example, Woebot? What opportunities do they offer and what are their limitations?
- How could they influence the doctor – patient relationship? In your opinion, what would chatbots need to do in order for both patients and healthcare professionals to benefit from them in a meaningful way?
- Which aspects of the use of chatbots in medicine do you see as particularly critical and why?
- How do you think a chatbot should be used in terms of dialog design? How relevant are factors such as emoji use, artificial delays, language and small talk?
- What aspects should be considered in terms of linguistic style? Should the dialog be designed differently regardless of the target group?
- How would you describe the optimal personality of the chatbot? Do you think a chatbot should have personality and/or show emotion? How far can a chatbot be humanized in your view?

### A2. I have a positive attitude towards the application and development of chatbots in medicine, because…

**Table:**

Item		Agree entirely	Rather agree	Undecided	Rather disagree	Disagree entirely	Mean (SD) Prä	Wilcoxon *p* < 0.05
I believe that the use of chatbots brings more opportunities than risks.	Prä	8.3%	41.7%	33.3%	16.7%	0%	3.4	n.s.
		(1/12)	(5/12)	(4/12)	(2/12)	(0/12)	(0.9)	
	Post	27.3%	18.2%	45.5%	9.1%	0%	3.7	
		(3/11)	(2/11)	(5/11)	(1/11)	(0/11)	(1.0)	
you save time by using chatbots	Prä	25.0%	58.3%	16.7%	0%	0%	4.1	n.s.
		(3/12)	(7/12)	(2/12)	(0/12)	(0/12)	(0.7)	
	Post	45. 5%	27.3%	18.2%	9.1%	0%	4. 1	
		(5/11)	(3/11)	(2/11)	(1/11)	(0/11)	(1.0)	
you save money by using chatbots	Prä	33.3%	41.7%	25.0%	0%	0%	4.1	n.s
		(4/12)	(5/12)	(3/12)	(0/12)	(0/12)	(0.79)	
	Post	27.3%	27.3%	45.5%	0%	0%	3.8	
		(3/11)	(3/11)	(5/11)	(0/11)	(0/11)	(0.8)	
the integration of these, the participation in therapy decisions of patients increases (e.g., less language barriers)	Prä	16.7%	41.7%	25.0%	0%	8.3%	3.6	n.s
		(2/12)	(5/12)	(3/12)	(0/12)	(1/12)	(1.1)	
	Post	9.1%	36.4%	45.5%	9.01%	0%	3.5	
		(1/11)	(4/11)	(5/11)	(1/11)	(0/11)	(0.8)	
Chatbots enable inclusion of people who otherwise have inhibitions about confiding in a doctor	Prä	8.3%	33.3%	33.3%	8.3%	16.7%	3.1	n.s
		(1/12)	(4/12)	(4/12)	(1/12)	(2/12)	(1.2)	
	Post	18.2%	27.3%	45.5%	9.1%	0%	3.6	
		(2/11)	(3/11)	(5/11)	(1/11)	(0/11)	(0.9)	
Chatbots are available to everyone regardless of time and location	Prä	33.3%	58.3%	8.3%	0%	0%	4.3	n.s
		(4/12)	(7/12)	(1/12)	(0/12)	(0/12)	(0.6)	
	Post	54.6%	36.4%	9.1%	0%	0%	4.5	
		(6/11)	(4/11)	(1/11)	(0/11)	(0/11)	(0.7)	
Chatbots can draw on a large database and thus have access to more knowledge than a physician	Prä	50.0%	25.0%	16.7%	8.3%	0%	4.2	n.s
		(6/12)	(3/12)	(2/12)	(1/12)	(0/12)	(1.0)	
	Post	36.4%	27.3%	27.3%	9.1%	0%	3.9	
		(4/11)	(3/11)	(3/11)	(1/11)	(0/11)	(1.0)	
Chatbots are neutral listeners without being personally judgmental	Prä	16.7%	8.3%	41.7%	16.7%	16.7%	2.3	n.s
		(2/12)	(1/12)	(5/12)	(2/12)	(2/12)	(1.3)	
	Post	9.1% (1/11)	36.4% (4/11)	27.3% (3/11)	27.3% (3/11)	0% (0/11)	3.3 (1.0)	

### A3. I am critical of the use of chatbots in medicine, because…

**Table:**

Item		Agree entirely	Rather agree	Undecided	Rather disagree	Disagree entirely	Mean (SD)	Wilcoxon p < 0.05
I believe that data protection and data security cannot be fully guaranteed	Prä	16.7%	33.3%	41.7%	0%	8.3%	3.5	0.02
		(2/12)	(4/12)	(5/12)	(0/12)	(1/12)	(1.1)	
	Post	0%	36.4%	18.2%	27.3%	18.2%	2.7	
		(0/11)	(4/11)	(2/11)	(3/11)	(2/11)	(1.14)	
the health data can be easily manipulated	Prä	16.7%	33.3%	25.0%	16.7%	8.3%	3.3	n.s.
		(2/12)	(4/12)	(3/12)	(2/12)	(1/12)	(1.2)	
	Post	9.1%	36.4%	18.2%	18.2%	18.2%	3.0	
		(1/11)	(4/11)	(2/11)	(2/11)	(2/11)	(1.3)	
the personal contact gets lost	Prä	50.0%	16.7%	25.0%	8.3%	0%	4.1	n.s
		(6/12)	(2/12)	(3/12)	(1/12)	(0/12)	(1.1)	
	Post	27.3%	36.4%	9.1%	27.3%	0%	3.6	
		(3/11)	(4/11)	(1/11)	(3/11)	(0/11)	(1.15)	
I am not ready to discuss sensitive data with a chatbot.	Prä	25.0%	16.7%	25.0%	16.7%	16.7%	3.2	n.s
		(3/12)	(2/12)	(3/12)	(2/12)	(2/12)	(1.5)	
	Post	36.4%	9.1%	9.1%	36.4%	9.1%	3.3	
		(4/11)	(1/11)	(1/11)	(4/11)	(1/11)	(1.5)	
there may be possible communication problems due to immature technology	Prä	50.0%	50.0%	0%	0%	0%	4.5	n.s
		(6/12)	(6/12)	(0/12)	(0/12)	(0/12)	(0.5)	
	Post	54.6%	27.3%	18.2%	0%	0%	4.4	
		(6/11)	(3/11)	(2/11)	(0/11)	(0/11)	(0.8)	
Chatbots are not established enough yet and long-term success is yet to come	Prä	25.0%	33.3%	33.3%	8.3%	0%	3.8	n.s
		(3/12)	(4/12)	(4/12)	(1/12)	(0/12)	(1.0)	
	Post	36.4%	45.5%	18.2%	0%	0%	4.2	
		(4/11)	(5/11)	(2/11)	(0/11)	(0/11)	(0.7)	
I am concerned that my data will be transferred and evaluated from uninvolved third parties	Prä	16.7%	50.0%	16.7%	8.3%	8.3%	3.6	n.s
		(2/12)	(6/12)	(2/12)	(1/12)	(1/12)	(1.2)	
	Post	18.2%	9.1%	18.2%	36.4%	18.8%	2.8	
		(2/11)	(1/11)	(2/11)	(4/11)	(2/11)	(1.4)	
